# Supporting the adaptive capacity of species through more effective knowledge exchange with conservation practitioners

**DOI:** 10.1111/eva.13266

**Published:** 2021-07-06

**Authors:** Carly N. Cook, Erik A. Beever, Lindsey L. Thurman, Laura M. Thompson, John E. Gross, Andrew R. Whiteley, Adrienne B. Nicotra, Jennifer A. Szymanski, Carlos A. Botero, Kimberly R. Hall, Ary A. Hoffmann, Gregor W. Schuurman, Carla M. Sgrò

**Affiliations:** ^1^ School of Biological Sciences Monash University Clayton VIC Australia; ^2^ Northern Rocky Mountain Science Center U.S. Geological Survey Bozeman MT USA; ^3^ Department of Ecology Montana State University Bozeman MT USA; ^4^ Northwest Climate Adaptation Science Center U.S. Geological Survey Corvallis OR USA; ^5^ National Climate Adaptation Science Center U.S. Geological Survey Reston VA USA; ^6^ Department of Forestry, Wildlife and Fisheries University of Tennessee Knoxville TN USA; ^7^ Climate Change Response Program U.S. National Park Service Fort Collins CO USA; ^8^ Wildlife Biology Program Department of Ecosystem and Conservation Sciences Franke College of Forestry and Conservation University of Montana Missoula MT USA; ^9^ Division of Ecology and Evolution Research School of Biology Australian National University Canberra ACT Australia; ^10^ Division of Endangered Species U.S. Fish and Wildlife Service La Crosse WI USA; ^11^ Department of Biology Washington University St Louis MO USA; ^12^ The Nature Conservancy, North America Region Haslett Michigan USA; ^13^ School of BioSciences Bio21 Institute The University of Melbourne Melbourne VIC Australia

**Keywords:** adaptive capacity, conservation management, evidence‐based conservation, evolutionary adaptive capacity, knowledge exchange, natural resource management

## Abstract

There is an imperative for conservation practitioners to help biodiversity adapt to accelerating environmental change. Evolutionary biologists are well‐positioned to inform the development of evidence‐based management strategies that support the adaptive capacity of species and ecosystems. Conservation practitioners increasingly accept that management practices must accommodate rapid environmental change, but harbour concerns about how to apply recommended changes to their management contexts. Given the interest from both conservation practitioners and evolutionary biologists in adjusting management practices, we believe there is an opportunity to accelerate the required changes by promoting closer collaboration between these two groups. We highlight how evolutionary biologists can harness lessons from other disciplines about how to foster effective knowledge exchange to make a substantive contribution to the development of effective conservation practices. These lessons include the following: (1) recognizing why practitioners do and do not use scientific evidence; (2) building an evidence base that will influence management decisions; (3) translating theory into a format that conservation practitioners can use to inform management practices; and (4) developing strategies for effective knowledge exchange. Although efforts will be required on both sides, we believe there are rewards for both practitioners and evolutionary biologists, not least of which is fostering practices to help support the long‐term persistence of species.

## 1 INTRODUCTION

Unprecedented rates of environmental change have already led to significant impacts on the natural world (Díaz et al., 2019; Scheffers et al., 2016). The extent and pace of these changes mean that many species face extinction if they are not able to tolerate or adjust to changes *in situ*, shift their distributions to track more favourable conditions or evolve in response to changing conditions (Dawson et al., 2011; Quintero & Wiens, 2013). The potential to facilitate adaptation to climate change has stimulated interest in research identifying management strategies intended to reduce or ameliorate the impacts of environmental changes and strategies to enhance or support species’ adaptive capacity (e.g. Beever et al., 2016; Prober et al., 2019). Adaptive capacity (Box 1) refers to the intrinsic ability of species to cope with or adjust to changing environmental conditions, including via genetic (i.e. evolutionary potential; Box 1) and/or phenotypic changes (phenotypic plasticity; Box 1).

BOX 1 **Glossary of terms**

*Adaptive capacity*: the ability of species or populations to intrinsically cope with or adjust to environmental change, either through genetic changes, phenotypic plasticity and/or dispersal.

*Adaptive management*: a structured and iterative process for decision‐making that has the goal of reducing uncertainty over time, via monitoring and updating knowledge as part of ongoing learning.

*Assisted colonization*: human relocation of individuals to sites where the species does not currently occur or has not been known to occur in recent history. A sub‐category of ‘assisted migration’.

*Assisted gene flow*: the intentional translocation of individuals within the range of a species to facilitate adaptation to anticipated local conditions. Also called ‘gene‐pool mixing’.

*Assisted migration*: the practice of moving plants or animals to new locations either within or outside of their species range, often to match organisms with their historic climates as global warming occurs. Also termed ‘managed relocation’, ‘assisted dispersal’, ‘assisted colonization’.

*Boundary organization*: an institution that plays an intermediary role between scientists and decision‐makers, including by commissioning research, facilitating communication between scientists and practitioners, and translating existing evidence into context‐specific knowledge.

*Evolutionary potential*: the capacity for populations to respond to selection pressures through genetic changes. Also referred to as ‘evolutionary adaptive capacity’ or ‘adaptive potential’.

*Genetic rescue*: restoring gene flow into small, isolated population to reverse negative fitness consequences.

*Knowledge broker*: an individual who plays an intermediary role between scientists and decision‐makers, assisting scientists to understand the management context and research needs of practitioners and assisting practitioners to interpret the relevant science for their management contexts.

*Knowledge co*‐*production*: a collaborative process to generating knowledge that involves practitioners and scientists working together from a project's inception to frame the problem, generate context‐specific knowledge and identify pathways to achieve objectives.

*Phenotypic plasticity*: the range of phenotypes (the physical expression of genotypes) that a given genetic individual can express as a function of environment.

*Risk assessment framework*: a decision support process that identified and evaluates the factors that influence the chance of positive or negative outcomes associated with a management action.

*Translational science*: intentional processes in which scientists, stakeholders, and decision‐makers work together to develop ecological research via joint consideration of the social, ecological, and political contexts of a problem.

Interest in using conservation management practices to support the adaptive capacity of species has driven attempts to distil the evidence base evaluating the conditions and contexts that promote or inhibit adaptation (e.g. Hoffmann & Sgrò, 2011; Nicotra et al., 2015; Stockwell et al., 2003) and the attributes, traits and conditions that influence the levels of tolerance and degree of flexibility species display in their responses to change (e.g. Beever et al., 2017; Foden et al., 2013; Mimura et al., 2017). These efforts in turn have led to frameworks to evaluate the adaptive capacity of species (e.g. Thurman et al., 2020), and a proliferation of recommendations for actions and approaches to support species’ responses to climate change (e.g. Broadhurst et al., 2017; LeDee et al., 2021).

Despite the theoretical evidence base amassed by documenting the processes by which species respond to change (Mimura et al., 2017; Stockwell et al., 2003), and the increasing number of management recommendations (Carroll et al., 2014; Crandall et al., 2000; Hendry et al., 2011; Smith et al., 2014) and decision support frameworks (e.g. Frankham et al., 2011; Hoffmann et al., 2021; Prober et al., 2015; Weeks et al., 2011), conservation practitioners (i.e. policy‐makers and on‐ground managers) remain uncertain about how to manage for the adaptive capacity of species and populations (Cook & Sgrò, 2018; Thurman et al., 2020). Particular areas of concern include how to determine if, when and where adaptive capacity is sufficient for a species to reduce extinction risk, how to recognize when a lack of adaptive capacity might be a risk for species and how to identify appropriate management actions to facilitate adaptation (Cook & Sgrò, 2018). These areas of apprehension point to the gap between the available science and the ability of practitioners to integrate this evidence into their management decisions; this gap constitutes a major barrier to achieving the required advancements to conservation practices (Beier et al., 2017).

The gap between science and practice when facilitating adaptation to climate change is emblematic of a broader problem of translating evolutionary biology into conservation policy and practice (Carroll et al., 2014; Cook & Sgrò, 2017; Kinnison et al., 2007; Smith et al., 2014). For decades, evolutionary biologists have been highlighting how evolutionary theory can inform effective conservation practices (e.g. Carroll et al., 2014; Crandall et al., 2000; Frankel & Soule, 1981; Frankham, 2005; Futuyma, 1995; Hendry et al., 2011; Smith et al., 2014). However, progress has been slow in changing conservation practices to account for evolutionary principles and processes (Cook & Sgrò, 2018). Meanwhile, evolutionary biologists continue to warn that well‐established management practices may do more harm than good (e.g. treating fragmented populations as separate management units, Bell et al., 2019; Weeks et al., 2016; or prioritizing local seed provenances in restoration, Broadhurst et al., 2008; Prober et al., 2015) and that there is a need to change prevailing management paradigms (Prober et al., 2019; Ralls et al., 2018).

Practitioners report a range of barriers to modifying their management practices, many of which could be addressed through better collaboration between evolutionary biologists and practitioners (Cook & Sgrò, 2018; Ridley & Alexander, 2016). Building on existing efforts to translate evolutionary theory into practical guidelines for management (e.g. Frankham et al., 2011; Hoffmann et al., 2021; Prober et al., 2015; Sgrò et al., 2011), we believe progress could be accelerated by drawing on lessons for successful knowledge exchange that have been developed by other disciplines (Cook et al., 2013; Enquist et al., 2017; Norström et al., 2020). Facilitating evidence‐based decisions requires tools and strategies that foster collaboration between scientists and practitioners, increase access to credible and relevant evidence (Sutherland et al., 2004), support practitioners to interpret evidence for their management context (Cook et al., 2013) and promote the two‐way communication needed for practitioners and researchers to learn from one another (Norström et al., 2020; Ridley & Alexander, 2016; Safford et al., 2017). These tools and strategies have been developed over decades by large, transdisciplinary communities of practice working to bridge the gap between science and practice and promote the integration of evidence into management decisions.

As a collective of conservation and evolutionary biologists and practitioners, our goal for this paper is to detail how general lessons from knowledge exchange can be used to support better integration of evolutionary theory into conservation management, with a particular focus on supporting the adaptive capacity of species and populations. These lessons include the following: (1) recognizing why practitioners do and do not use evidence; (2) considering how to build an evidence base that will influence management decisions; (3) understanding how to translate evolutionary theory into a form that conservation practitioners can use to inform management practices; and (4) developing strategies for effective knowledge exchange to support the required changes to management.

## 2 LESSON 1 – RECOGNIZING WHY PRACTITIONERS DO AND DO NOT USE SCIENTIFIC EVIDENCE

**FIGURE 1:**
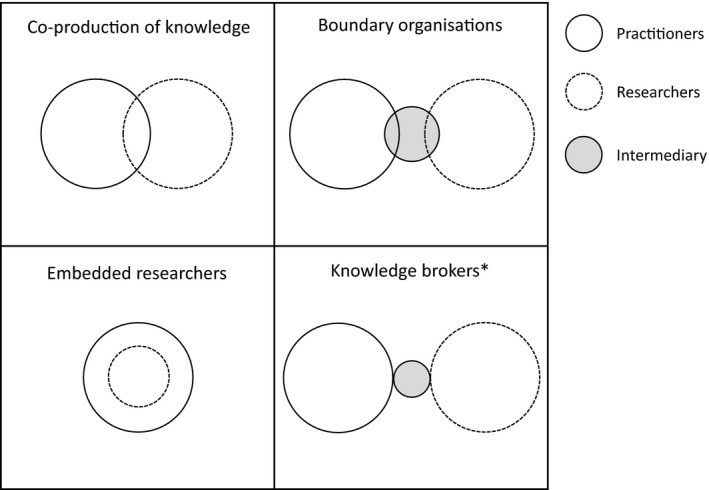
Models of knowledge exchange that depict the relationships amongst practitioners, researchers and through individuals (knowledge brokers) or groups (boundary organizations) that act as intermediaries. *Knowledge brokers can be embedded within research teams or independent organizations. Adapted from Cvitanovic et al. (2015)

Research seeking to understand why a gap exists between conservation science and practice has revealed a wide range of barriers to the use of scientific evidence (Cvitanovic et al., 2015; Esler et al., 2010; Fabian et al., 2019; Habel et al., 2013). Many of these barriers revolve around practitioners being able to access relevant and credible evidence and apply it to their management context (Walsh et al., 2019); challenges practitioners face regardless of the scientific discipline (Jørgensen et al., 2019). In response, many conservation journals are now open access and have explicit objectives to promote practice‐oriented research with strong stakeholder participation (e.g. *Conservation Science and Practice*, Schwartz et al., 2019; *Ecological Solutions and Evidence*, Cadotte et al., 2020). By publishing research in open‐access journals whose audiences include practitioners (e.g. *Evolutionary Applications*), evolutionary biologists can extend the reach of their research. Although practitioners may not always have the time or capacity to access the primary literature themselves, better access to management‐relevant primary research with clear recommendations for conservation practice can assist those in boundary‐spanning roles (e.g. knowledge brokers, boundary organizations, researchers embedded in management agencies; Figure 1, Box 1) to facilitate reciprocal knowledge exchange between scientists and decision‐makers (see Lesson 4).

Another barrier to the use of scientific evidence by practitioners is the mismatch between the research conducted (e.g. documenting threats) and the knowledge practitioners need for decision‐making (e.g. identifying effective solutions), even for highly applied disciplines like restoration ecology and invasion biology (Beever et al., 2019; Esler et al., 2010; Fazey et al., 2005; Young & Van Aarde, 2011). Research aimed at encouraging practitioners to support species’ adaptive capacity must likewise transition from documenting the negative impacts of climate change on species and ecosystems (Scheffers et al., 2016) to investigating the effectiveness of proposed management strategies to increase evolutionary potential (e.g. Prober et al., 2015; Weeks et al., 2017; Box 1). Achieving the latter includes reporting on climate adaptation experiments that test the efficacy of management interventions in promoting species’ adaptive capacity and their responses to contemporary global change. Recommendations derived from experimental studies of model organisms, conducted under controlled conditions, are unlikely to persuade practitioners to alter long‐standing management approaches (Cook & Sgrò, 2018). Instead, working with practitioners to support adaptive management (Box 1) or to involve them in research that demonstrates how changing management practices will lead to better conservation outcomes will provide more compelling evidence (see Lesson 2).

A commonly perceived barrier to the use of scientific evidence in conservation is that practitioners are apprehensive about interpreting studies written for academic audiences (Sunderland et al., 2009). This disinclination likely constitutes a significant problem for two‐way communication between evolutionary biologists and conservation practitioners, given that the two groups have lexicons that include a litany of terms that are neither intuitive nor always consistently defined (Ridley & Alexander, 2016). Conservation practitioners report that evolutionary principles are often not included within traditional natural resource management curricula (Cook & Sgrò, 2018) and that they have little training in how to apply principles from evolution and genetics to their management decisions (Cook & Sgrò, 2019). Insufficient exposure to evolutionary theory can make it difficult for practitioners to identify and critically evaluate relevant research and to determine how it can be applied to various management situations. Concepts such as adaptive capacity are multifaceted (Thurman et al., 2020), which makes it challenging for practitioners to operationalize broad recommendations. Therefore, to promote changes to management practice, evolutionary biologists need to collaborate with practitioners to build their knowledge of important evolutionary concepts (Carroll et al., 2014) and ensure that research findings are clearly articulated and directly applicable to management contexts (Beier et al., 2017; Enquist et al., 2017; Ridley & Alexander, 2016).

Beyond the challenges mentioned above, there are pragmatic reasons why scientific evidence may not be used in decision‐making. Management decisions occur within social, political and economic contexts, which can restrict practitioners’ ability to implement best practices (Norström et al., 2020; Sutherland et al., 2004). Social and political constraints on management decisions can be an impediment to managing for adaptive capacity, wherein for example, assisted gene flow (Box 1) and assisted migration (Box 1) are controversial management strategies (Aitken & Whitlock, 2013; Lawler & Olden, 2011; Schwartz et al., 2012). There are also legislative and regulatory barriers to implementing some strategies, such as listing sub‐species, distinct population segments or ‘management units’ as separate entities for protection (Weeks et al., 2016) and hybrids having uncertain legal status (Chan et al., 2019). Other practical barriers include spatial mismatches, wherein place‐based management occurs at local geographic scales (e.g. protected areas), yet adaptive capacity needs to be managed at broader landscape scales, often across jurisdictional boundaries and different land tenures (Beever et al., 2014; Cook & Sgrò, 2018). Working with practitioners, evolutionary biologists can better understand the socio‐ecological contexts and practical constraints on management decision and propose solutions that can be implemented within realistic management settings (Enquist et al., 2017; Fazey et al., 2005; Folke et al., 2005; Norström et al., 2020). For example, although the costs of genomic data continue to fall (Breed et al., 2019), genetic monitoring across multiple populations, over several generations will only be feasible for a small number of species. Overcoming barriers to managing for adaptive capacity may require evolutionary biologists to also engage with high‐level policy‐makers and key stakeholders, as conservation biologists have often done (e.g. reintroducing keystone species: grey wolves in Yellowstone; Smith et al., 2003), to help shape the political landscape for practitioners.

## 3 LESSON 2 – BUILDING AN EVIDENCE BASE THAT WILL INFLUENCE MANAGEMENT DECISIONS

**FIGURE 2:**
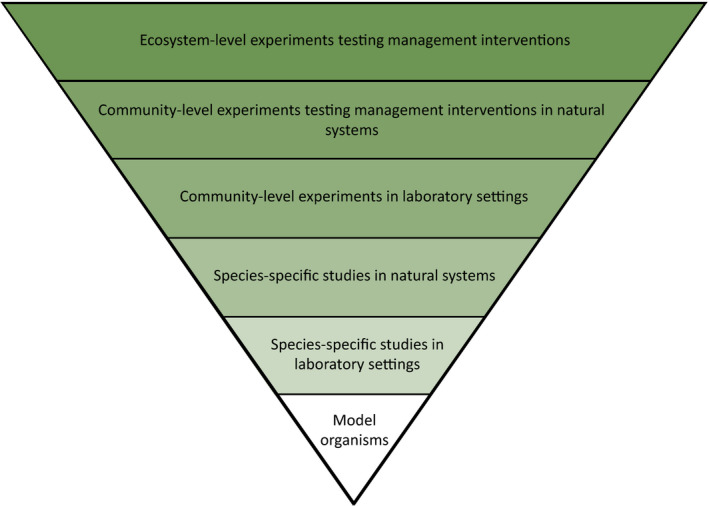
A robust evidence base to support successful management practices, based on an incremental scaling up in spatial extent and ecological complexity, from studies of model organisms to studies documenting changes in whole ecosystems

In calling for practitioners to manage the adaptive capacity of species, evolutionary biologists are proposing a major shift from a default position of ‘no action’ to a more proactive approach of actively managing genetic diversity (Chan et al., 2019; Ralls et al., 2018; Stockwell et al., 2003). For example, practitioners have typically been taught that gene‐pool mixing can disrupt local adaptation and erode genetic uniqueness (Moritz, 1999), but are learning that wholesale avoidance of this approach may compromise the persistence of small, fragmented populations (Weeks et al., 2016; Whiteley et al., 2015). Conservation practitioners harbour concerns about the potential negative outcomes associated with actively managing genetic diversity (e.g. outbreeding depression, disease risk; Cook & Sgrò, 2018) and want to understand the contexts in which strategies like genetic rescue (Box 1) and assisted migration will yield benefits—questions without easy answers (Bell et al., 2019; Ridley & Alexander, 2016; Tallmon et al., 2004). It is therefore necessary to work with practitioners to provide clear guidance for the circumstances under which current management practices are harmful (Prober et al., 2015; Weeks et al., 2016), and when new approaches will achieve better conservation outcomes (Bell et al., 2019; Etterson et al., 2020; Frankham, 2016; Weeks et al., 2017). Although considerably more difficult than for laboratory‐based studies, building an evidence base that demonstrates benefits outside of model organisms, over multiple generations, in natural systems and under realistic management conditions (e.g. 35‐year provenance trials; Prober et al., 2015), is important to ensure evidence is fit‐for‐purpose (Figure 2).

To date, much of the evidence for the benefits of actively managing genetic diversity in natural systems has come from a small but growing number of case studies (e.g. Bell et al., 2019; Chan et al., 2019; Weeks et al., 2017). Case studies provide essential pieces of evidence, but the implementation (e.g. amount of gene flow, degree of population structure, distance moved), and associated outcomes are often context‐ and taxon‐specific, making them difficult to generalize. Furthermore, assisted gene flow is often employed as a last‐ditch attempt to prevent extinction of severely inbred populations (e.g. genetic rescue of *Burramys parvus*; Weeks et al., 2017) rather than as part of a forward‐looking strategy to build adaptive capacity. These types of case studies do not necessarily offer lessons to guide proactive management efforts to prevent the loss of genetic diversity. Alongside threats with significant but longer‐term consequences (e.g. shifting climate envelopes), practitioners are also generally managing multiple threats simultaneously (e.g. habitat loss, invasive predators; Díaz et al., 2019; Legge et al., 2018), often with severe and immediate consequences for populations. Therefore, it can be difficult for practitioners to determine when and how recommendations for managing evolutionary potential should be applied to their context (Hendry et al., 2010). Consequently, evolutionary biologists have developed generalized risk assessment frameworks (e.g. Frankham et al., 2011; Box 1) and rules of thumb (e.g. Hoffmann et al., 2021; Sgrò et al., 2011; Weeks et al., 2011) to assist practitioners, but generalizations also come with important caveats (e.g. measures of neutral versus adaptive diversity; Sgrò et al., 2011). Although an important step forward, practitioners require support to apply these frameworks to their decision contexts (Cook & Sgrò, 2019; see Lesson 4). More field studies that evaluate and expand decision support frameworks, and identify the factors or contexts that most strongly mediate the outcomes of a strategy to support adaptive capacity, could also prove helpful for practitioners.

Researchers often struggle to help practitioners make sense of apparently contradictory scientific findings. To help address this challenge, several fields, including health sciences, social welfare, education (Davies & Boruch, 2001), have adopted approaches to synthesize existing information generated by individual studies or reviews, to identify which practices work well and under what circumstances (Dicks et al., 2014; Pullin & Knight, 2001). Identifying and collating relevant studies that evaluate the effectiveness of management interventions under a wide range of contexts can be used to assess overall patterns (Pullin et al., 2016). There are many approaches to evidence synthesis, some involving meta‐analysis (Gurevitch et al., 2001) to assess the impact of effect modifiers on the effectiveness of an intervention, and some using more qualitative assessments of the weight of evidence (Cook et al., 2017). The value of this strategy has been demonstrated by conservation practitioners being willing to change their management practices when presented with a synthesis of the available evidence base (Walsh et al., 2015). Therefore, increasing the number of individual studies that address questions of when and how to promote adaptive capacity, conducted under a wide range of realistic conditions, would help build an evidence base that could support widespread changes to management practices.

Growing the evidence base for best‐practice management of adaptive capacity could be assisted by helping practitioners formulate interventions as hypotheses and to implement management actions as an experiment (e.g. adaptive management, Walters & Holling, 1990) or at least supporting them to document the outcomes (both positive and negative) of management actions (Margoluis et al., 2009). Adaptive management is used in other fields to manage uncertainty and learn from management activities in order to update future strategies (Folke et al., 2005; Jørgensen et al., 2019; Williams & Brown, 2014). These types of management experiments have been used to document strategies to promote adaptive capacity (e.g. landscape restoration projects and seed provenance trials; Broadhurst et al., 2008). This approach would be particularly useful in identifying when a lack of adaptive capacity may be a threat to long‐term persistence, and whether actions to promote adaptive capacity have been successful. Working closely with practitioners in this way can be critical, given that management decisions tend to occur over shorter timescales than required to demonstrate the benefits of supporting adaptive capacity (Prober et al., 2019), although not always (e.g. Hendry et al., 2011; Weeks et al., 2017).

## 4 LESSON 3 – TRANSLATING THEORY INTO A FORMAT THAT CONSERVATION PRACTITIONERS CAN USE TO INFORM MANAGEMENT PRACTICES

Evolutionary theory can provide insights into the characteristics that influence a species’ ability to respond to selective forces, which are relevant to conservation management (Carroll et al., 2014; Hoffmann & Sgrò, 2011). A key challenge is packaging those insights so they effectively inform management practices (Enquist et al., 2017; Safford et al., 2017). A robust theoretical foundation may be perceived by researchers as compelling evidence, but practitioners may not appreciate the caveats and context‐dependencies that determine when applying these general rules is appropriate (Beier et al., 2017). This uncertainty can be paralysing for practitioners tasked with interpreting general rules (Tallmon et al., 2004).

In attempting to bridge the gap between science and practice, evolutionary biologists must walk a difficult line in ensuring advice is sufficiently general to be applied widely (i.e. risk assessment frameworks; Frankham et al., 2011), while also recognizing and accounting for the complex realities of local conditions (Aitken & Whitlock, 2013; Bell et al., 2019; Hendry, 2016). Rules of thumb (e.g. adaptive diversity is associated with environmental gradients; Sgrò et al., 2011) and generalized risk assessments (e.g. probability of outbreeding depression; Frankham et al., 2011) can be of great value in determining when to consider supporting adaptive capacity. How to implement these rules in practice is a more nuanced question that practitioners may need support to answer. For example, the general rule that one migrant per generation will maintain genetic diversity assumes that the migrant successfully reproduces (Wang, 2004). Likewise, maintaining an effective population size (*N*_e_) of >1000 to maintain evolutionary potential (Frankham et al., 2014) requires understanding the relationship between *N* and *N*_e_, which can vary widely amongst species and across different environments and the spatial scale at which *N*_e_ is evaluated (Frankham, 1995; Palstra & Fraser, 2012).

With a proliferation of management recommendations (Crandall et al., 2000; Hendry et al., 2011; Kinnison et al., 2007; Stockwell et al., 2003), the risk is that practitioners assume advice can only be provided on the basis of extensive data and that management problems require bespoke solutions. Practitioners need to know when rules of thumb can be applied, and when additional data are required to make decisions. The advice that genomic data can help tailor recommendations to specific management contexts (Breed et al., 2019) needs to be accompanied by caveats around the acquisition and analysis of genomic data. For instance, distinguishing between neutral and adaptive diversity in genomic data requires associated fitness data (Hoffmann et al., 2015) to understand the genetic differences that reflect the diversity on which selection can act, rather than those that may be signatures of genetic drift (Weeks et al., 2016). Guidelines are lacking that can help practitioners understand when genomic data are required to inform decisions, and how to collect and use these data to develop strategies to support evolutionary potential. This is where two‐way communication between scientists and practitioners can help to formulate the questions that need to be addressed (Enquist et al., 2017; Ridley & Alexander, 2016). Other decision support tools used in conservation, such as structured decision‐making and adaptive management (reviewed in Schwartz et al., 2018), but tailored to evolutionary challenges, could make a significant difference in supporting practitioners to accommodate uncertainty in decisions.

As the number of studies assessing strategies to support adaptive capacity increase, and evidence documenting management experiments grows, synthesizing that evidence base can reveal when rules of thumb apply and the contexts in which alternative approaches are more effective (see Lesson 2). Although practitioners are willing to change their practices on the basis of new evidence (Walsh et al., 2015), there is no one‐size‐fits‐all approach to evidence‐based decision‐making (Cook et al., 2017). Issues of credibility and legitimacy can be highly influential in the evidence practitioners trust and use, making relationships between scientists and practitioners important for successful knowledge exchange (Beier et al., 2017; Cook et al., 2013; Enquist et al., 2017).

## 5 LESSON 4 – DEVELOPING STRATEGIES FOR EFFECTIVE KNOWLEDGE EXCHANGE

**FIGURE 3:**
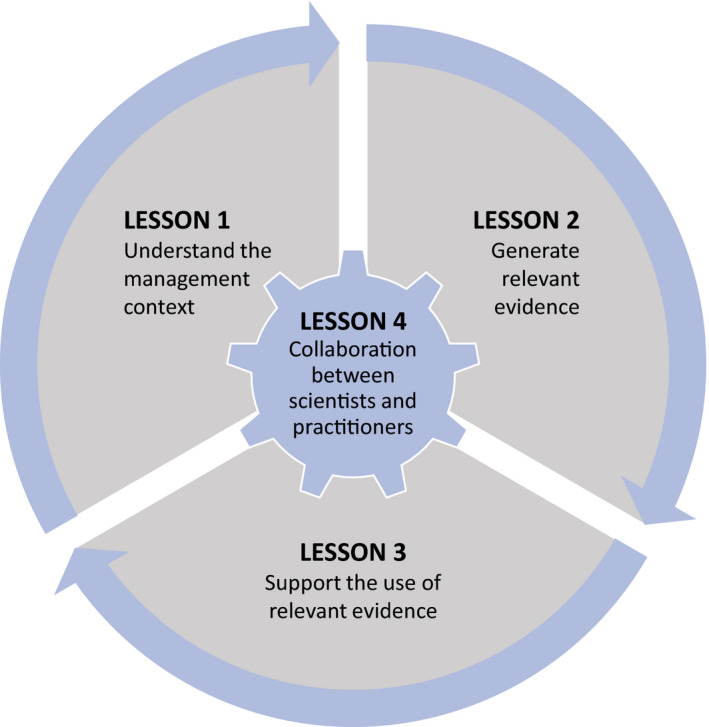
Four elements involved in supporting effective knowledge exchange. Collaboration between scientists and practitioners (Lesson 4) is essential to understanding the management context for a decision (Lesson 1), generating relevant evidence (Lesson 2) and supporting its use in decision‐making (Lesson 3). See Panel 1 for further details

Changing management paradigms is not easy, and practitioners face significant environmental challenges and limited resources. Management strategies are often reactive, focussed on short‐term outcomes (e.g. suppressing threats), whereas longer‐term outcomes can require more proactive strategies that promote functional and resilient ecosystems. Supporting the adaptive capacity of species requires transitioning away from such reactive management strategies (e.g. addressing inbreeding depression in critically endangered species; Bell et al., 2019) to more proactive approaches (e.g. facilitating adaptive capacity by preventing the erosion of genetic diversity; Prober et al., 2019). These shifts require more than publishing research findings in open‐access journals (Lesson 1) or synthesizing evidence into broader recommendations (Lesson 3). Practitioners need to be directly supported by evolutionary biologists to make the necessary changes (Cook & Sgrò, 2018). This type of support can be provided via multiple strategies that facilitate the integration of evidence into practice (Cvitanovic et al., 2015; Figure 1), which are important for the success of the other lessons we outline here (Figure 3).

Two‐way knowledge exchange between scientists and practitioners is integral to evidence‐based decision‐making, because it increases the capacity of practitioners to understand the science, and builds the capacity of scientists to comprehend management challenges and contexts needed to formulate effective recommendations (Cook et al., 2013; Enquist et al., 2017; Norström et al., 2020; Panel 1). The benefits of engaging with practitioners to conduct management experiments extend beyond building the evidence base (Lesson 2), but can also offer researchers opportunities to conduct field‐based studies and train early‐career researchers. Participatory, practice‐oriented research that creates management‐research alliances (e.g. co‐production of knowledge; Figure 1, Box 1) enable scientists and practitioners to cooperatively develop effective strategies to address critical management challenges (Cvitanovic et al., 2015; Swart et al., 2014). Involving practitioners in formulating research questions can be a powerful way to draw on their knowledge about the systems they manage, understand the constraints on management decisions and ensure that proposed management alternatives can be critically evaluated and implemented (Beier et al., 2017).

**PANEL 1:**
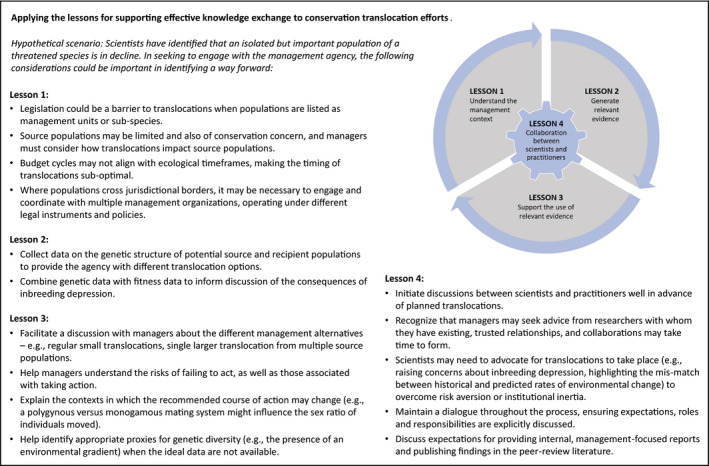
Applying the lessons for supporting effective knowledge exchange to conservation translocation efforts

Recent efforts to understand the uncertainties practitioners have about building adaptive capacity have revealed a range of questions for which the current evidence base cannot provide clear guidance (e.g. how transferable is knowledge about adaptive capacity across populations and related taxa?; Aitken & Whitlock, 2013, or can the evolutionary history of a species be used to predict its adaptive capacity?; Hendry et al., 2011). Determining what the real versus perceived knowledge gaps are for best‐practice management of adaptive capacity is an important first step (Beier et al., 2017). This is one area where individuals working at the interface of science and practice (e.g. knowledge brokers; Figure 1) and groups whose roles are to span the research‐action boundary (e.g. boundary organizations; Figure 1) can be critical (Hallett et al., 2017; Safford et al., 2017). Organizations, like the US Geological Survey's National and Regional Climate Adaptation Science Centers, can serve to identify knowledge gaps that require more research and fill perceived knowledge gaps by coordinating the co‐production of science and translating existing science into recommendations for practice. Similarly, initiatives like the EvolvES global research project (formerly bioGENESIS) within the Future Earth network (www.futureearth.org) promote applied evolutionary biology through research focused on questions relevant to biodiversity science and policy, and encouraging researchers to actively transmit their findings to policy‐makers (Hendry et al., 2010). Collaborative approaches can provide innovative funding sources, such as between the Wildlife Conservation Society Climate Adaptation Fund, funded by the Doris Duke Charitable Foundation. This programme has funded over 100 projects to implement and evaluate climate adaption actions, with project teams that often involve collaborations between practitioners and academic researchers (Cross et al., 2018).

Another strategy that can be highly effective at influencing management practices is to embed scientists within management agencies and conservation organizations (Figure 1), where they act as a bridge between science and practice (Cook et al., 2013). When scientists trained in evolutionary biology work from within agencies to help navigate the decision space, they can help build the capacity of practitioners and shape conservation policy within their institutions (Roux et al., 2019). We know of some very effective examples of embedding evolutionary biologists and population geneticists, who facilitate a culture of supporting management‐relevant research and integrating evolutionary theory into decision‐making. However, in many places this strategy is under‐utilized, with neither evolutionary biologists nor often ecologists employed within management agencies (Roux et al., 2019). As the pace of environmental change accelerates, and more radical approaches to management are required to conserve biodiversity, evolutionary biologists can help shape the conservation policies required to facilitate species’ adaptive capacity. To achieve actionable research, a strong emphasis on translational science (Box 1) that facilitates collaboration between scientists and practitioners is needed (Beier et al., 2017; Enquist et al., 2017).

## 6 CONCLUSIONS

Practitioners are open to changing their management practices to support the adaptive capacity of species, but they need help to achieve the required shift in accepted methods, policies and practices. Despite arguably having less of a history of engaging with conservation practitioners (Smith & Bernatchez, 2008), evolutionary biologists can help address existing information gaps and facilitate changes to management practices. There are well‐acknowledged challenges for incentivizing and funding translational science (Norström et al., 2020), although climate change is providing an imperative for funders, governments and nongovernment agencies whose missions are to support biodiversity (Cross et al., 2018; Enquist et al., 2017). The lessons from decades of efforts to bridge the gap between science and practice outlined here could support evolutionary biologists and practitioners to build the capacity of species and ecosystems to adapt to environmental change. Practical steps towards better engagement between evolutionary biologists and practitioners include working together to document the outcomes of management, conducting research in management‐relevant contexts, synthesizing evidence to identify general lessons and developing tools to help practitioners interpret when to apply those rules to their specific contexts. Effective knowledge exchange between evolutionary biologists and practitioners will require efforts on both sides and is essential to achieving the changes required to conserve biodiversity and the evolutionary processes generating it.

## CONFLICT OF INTEREST

None declared.

## Data Availability

There are no data associated with this manuscript.
